# Hemichorea as the Initial Presentation of Graves’ Disease

**DOI:** 10.7759/cureus.100906

**Published:** 2026-01-06

**Authors:** Marisa N Simoes, Alexandra N Araujo, Axel O Ferreira, Rui Araujo

**Affiliations:** 1 Critical Care Medicine, Hospital Pedro Hispano, Matosinhos, PRT; 2 Endocrinology, Hospital Pedro Hispano, Matosinhos, PRT; 3 Neurology, Hospital Pedro Hispano, Matosinhos, PRT

**Keywords:** critical care, medical intensive care unit (micu), movement disorders, neurology, thyroid related disease, treatment of hyperthyroidism, tremors

## Abstract

Chorea is a hyperkinetic movement disorder characterized by involuntary, spontaneous, irregular, purposeless movements that flow unpredictably from one body part to another. An overlap between movement disorders and neuroendocrine abnormalities is widely recognized; however, chorea is a rare complication of hyperthyroidism. In this case report, we present a case of unilateral upper limb chorea as a manifestation of hyperthyroidism. We describe a clinical case of a 21-year-old male who presented repeatedly to the emergency department (ED) with acute-onset, involuntary proximal movements of the left upper limb (LUL), arrhythmic in nature, without involvement of the face, trunk, or lower limbs (LLs). Neurological examination and investigations (cerebral magnetic resonance imaging, electroencephalogram, cerebral computed tomography angiography, and lumbar puncture) were unremarkable, except for the involuntary purposeless movements previously mentioned. During a subsequent admission for recurrent symptoms, the patient was diagnosed with acute appendicitis. Concurrent laboratory evaluation revealed thyrotoxicosis with suppressed TSH, elevated free T4 and T3, and positive TSH-receptor antibodies, confirming Graves’ disease. Surgical intervention was postponed due to the risk of thyroid storm, and the patient was admitted to the intensive care unit for stabilization and initiation of antithyroid therapy. Treatment with methimazole, propranolol, cholestyramine, hydrocortisone, and Lugol’s iodine led to normalization of thyroid function and complete resolution of hemichorea on follow-up, supporting a causal link to thyrotoxicosis. Hemichorea is rare in patients with hyperthyroidism, but it can be the first manifestation of the condition. This clinical case draws attention to an atypical presentation of Graves' disease and its challenges. This initial presentation may experience a delayed diagnosis, as hyperthyroidism is not typically the first consideration, and usually presents with other symptoms, such as palpitations, weight loss, heat intolerance, irritability, proptosis, and hyperhidrosis. Early recognition and proper diagnosis are essential, as treating the underlying disease may lead to rapid resolution of chorea and avoid unnecessary treatments.

## Introduction

Chorea is a hyperkinetic movement disorder characterized by involuntary, spontaneous, irregular, purposeless movements that flow unpredictably from one body part to another [1,2]. These movements result from dysfunction of the basal ganglia, particularly involving dopaminergic pathways within the striatum [1,2]. Chorea may present as generalized or segmental, with hemichorea often prompting investigation for structural, vascular, or metabolic etiologies [3].

The differential diagnosis of chorea is broad and includes neurodegenerative diseases, cerebrovascular events, autoimmune disorders, metabolic abnormalities, drug-induced syndromes, and endocrine conditions [2-4]. While non-ketotic hyperglycemia is a well-recognized metabolic cause, endocrine-related chorea is uncommon [5]. An overlap between movement disorders and neuroendocrine abnormalities is widely recognized, with movement disorders including tremor, myoclonus, dystonia, Parkinsonism, and, more rarely, chorea [6].

Hyperthyroidism-related chorea is a rare manifestation, accounting for fewer than 2% of reported chorea cases, and is most frequently associated with Graves’ disease [2,3,7]. The underlying pathophysiology remains incompletely understood but is thought to involve increased dopaminergic sensitivity, altered cerebral metabolism, and autoimmune-mediated effects on basal ganglia function [6,8].

Hemichorea as the initial and isolated manifestation of Graves’ disease is particularly rare and may lead to delayed diagnosis or misdiagnosis of primary neurological or functional disorders [1,2]. Early recognition is clinically important, as treatment of the underlying thyrotoxicosis often results in rapid symptom resolution [7,9]. We report a case of unilateral upper limb chorea as the presenting manifestation of Graves’ disease in a young adult male, highlighting diagnostic challenges and the importance of thyroid function testing in hyperkinetic movement disorders.

## Case presentation

We describe a clinical case of a 21-year-old male, with no previous medical history, who presented to the emergency department (ED) with an acute onset of involuntary movements, mostly tremors and paresthesia on the left hemibody. He also presented with accompanying symptoms of polyphagia and hyperhidrosis.

Clinical observation

During observation in the ED, the patient presented with a blood pressure of 113/87 mmHg, a heart rate of 93 bpm, and oxygen saturation of 96% on room air. The patient displayed involuntary proximal movements of the left upper limb (LUL), arrhythmic in nature, without involvement of the face, trunk, or lower limbs (LL). The patient reported a sense of painful hypoesthesia in the left limbs, without any other deficits.

During examination, the patient remained alert, oriented, and cooperative, with the following neurological examination: language: no deficits; speech: no dysarthria; visual fields: intact (tested by confrontation); eyes: midline; position, no ophthalmoparesis; pupils: Isocoric and reactive; cranial nerves: no palsies; motor examination: arm extension test revealed arrhythmic involuntary movements of the LUL, predominantly proximal, with some inconsistency during distractibility tests. Segmental strength evaluation showed reduced use of the left hemibody (Grade 4+); sensory examination: left-sided painful hypoesthesia, including the face; and coordination: no appendicular ataxia.

Throughout the evaluation, the patient presented with a sensory alteration followed by involuntary movements of the left hemibody.

The initial blood workup was as follows (Table 1).

**Table 1:** Blood workup analysis

Parameter	Value	Reference value
Hemoglobin	17.1 g/dL	13–18 g/dL
Leukocytes	9.6×10⁹/L	4.5–13.5 ×10⁹/L
Platelets	216×10⁹/L	150–400 ×10⁹/L
Creatinine	0.68 mg/dL	0.7–1.3 mg/dL
Urea	19.6 mg/dL	19–44 mg/dL
Sodium	141 mEq/L	135–145 mEq/L
Potassium	3.8 mEq/L	3.5–5.0 mEq/L
Chloride	103 mEq/L	98–107 mEq/L
Calcium	10.2 mg/dL	8.9–10 mg/dL
Lactate dehydrogenase	165 U/L	125–220 U/L
Albumin	4.8 g/dL	3.5–5.0 g/dL
Total proteins	6.8 g/dL	6.0–8.3 g/dL
Creatine kinase	79 U/L	30–200 U/L
C-reactive protein	0.08 mg/L	<5 mg/L

All other diagnostic evaluations (cerebral magnetic resonance imaging, electroencephalogram, cerebral computed tomography angiography, and lumbar puncture) were normal, suggesting no evidence of underlying neurological pathology (Table 2).

**Table 2:** Imaging, electroencephalogram, and lumbar puncture

Exam	Result
Cerebral magnetic resonance imaging	There are no gross changes in the signal or morphology of the brain parenchyma with any evident pathological significance. No deviations from the midline. The cerebrospinal fluid spaces are patent, with morphology and size appropriate for age. The cerebellar tonsils are in a normal position.
Cerebral computed tomography angiography	We do not observe cerebral hemorrhagic lesions. There are no obvious signs of acute infarcts with established hypodensity. No evident occlusions of the major cerebral arteries. We do not observe vascular malformations accessible to the method used. The vertebral and carotid arteries show no hemodynamically significant stenosis at the cervical level.
Electroencephalogram	The EEG trace during wakefulness is within normal limits.
Lumbar puncture	Clear and colorless appearance. The protein level was 26.6 mg/dL, glucose was normal at 54 mg/dL, and the cell count showed 1 white blood cell per microliter and 2 erythrocytes per microliter. No organisms were identified, and cytology revealed no malignant cells.

Due to the scarcity of clinical and non-clinical information, a functional neurological disorder was assumed. Therefore, the patient was discharged to the outpatient clinic for further evaluation, with a clonazepam prescription, to which the symptoms were partially responsive.

One week later, the patient presented again to the ED with abnormal involuntary movements of the left arm associated with abdominal pain in the right iliac fossa and was diagnosed with acute appendicitis, with a proposal for laparoscopic appendectomy. Workup showed leukocytosis 16,440/μL, similar thyroid function (Table 3), and the abdomen CT showed a normal cecal appendix with thickened walls. 

**Table 3:** Thyroid function laboratory parameters ED: emergency department, ICU: intensive care unit, TSH: thyroid-stimulating hormone, TPO: thyroid peroxidase

	Reference value	First ED visit	Second ED visit (D0)	ICU (D1)	ICU (D3)	ICU discharge (D5)	One month after ICU discharge
TSH (μU/mL)	0.35-4.94 uUI/mL	<0.01 uUI/mL	<0.01 uUI/mL	<0.01 uUI/mL	<0.01 uUI/mL	<0.01 uUI/mL	1.12 uUI/mL
Free T4 (ng/dL)	0.7-1.48 ng/mL	1.83 ng/mL	2.04 ng/mL	2.15 ng/mL	1.87 ng/mL	1.26 ng/mL	0.86 ng/mL
Free T3 (ng/mL)	1.71-3.71 pg/mL	--	2.63 pg/mL	7.27 pg/mL	2.9 pg/mL	3.29 pg/mL	3.15 pg/mL
Anti-thyroglobulin antibodies (UI/mL)	<4.11 UI/mL	--	15.3 UI/mL	--	--	--	--
Anti-TPO antibodies (UI/mL)	<5.61 UI/mL	--	5.5 UI/mL	--	--	--	--
TSH receptor antibodies (UI/L)	≤1.75 UI/L	--	--	--	--	--	2.42 UI/L

The pending thyroid function from previous observations in the ED evidenced a low TSH <0.01 μU/mL and a high free T4 1.83 ng/dL, compatible with hyperthyroidism/thyroxicosis. The preoperative study blood workup included a repeated thyroid function, which showed similar results as the previous study, with suppressed TSH, high free T4, high T3, positive TSH receptor antibodies, upper normal limit of thyroglobulin, positive anti-thyroglobulin antibodies, and negative for anti-TPO antibodies (Table 3). Given the risk of thyroid storm in a patient with untreated hyperthyroidism and the stability of the abdominal infection, the surgical approach was delayed for two days. The patient was admitted to the Intensive Care Unit (ICU), where he remained for four days, started on antithyroid medication, and optimised preoperative conditions. He was started on methimazole 5 mg eight-hourly, propranolol 40 mg six-hourly, cholestyramine 4g eight-hourly, hydrocortisone 100 mg 12-hourly, and Lugol's solution six drops eight-hourly.

On day 3 of the ICU stay, the patient underwent laparoscopic appendectomy, was administered balanced anaesthesia, and managed intraoperatively with hydrocortisone and esmolol. There were no complications throughout surgery, and remained in the ICU two days postoperatively with favourable progression. At discharge to the surgical ward, the patient denied palpitations, dizziness, hyperhidrosis, pain, nausea, vomiting, or other focal symptoms. He was maintained on methimazole, propranolol, cholestyramine, and hydrocortisone throughout the stay. 

Due to a favourable evolution, the patient was discharged from the ICU 5 days after admission. Throughout the hospital stay, he was followed by an Endocrinologist and consulted for an outpatient consult. On discharge, the patient sustained an improving thyroid function, as shown in Table 3. The patient was maintained on propranolol, adjusted to 40 mg once daily, methimazole 5 mg once daily, and a plan to wean off hydrocortisone. During the outpatient consult, review of the patient's records showed normal thyroid function test two years back with TSH 1.763 μU/mL. The thyroid ultrasound showed an “enlarged thyroid gland, hyperechoic and heterogeneous texture, but without nodular structures.”

The patient was tapered off all medications except for methimazole 7.5 mg once daily. Given the clinical improvement that coincided with treatment and a normalized thyroid function, the hemichorea was attributed to Graves' disease.

## Discussion

This case illustrates a rare neurological presentation of Graves’ disease in which hemichorea preceded the development of classical systemic features of hyperthyroidism. The unilateral distribution of symptoms, associated sensory disturbances, and normal neuroimaging contributed to initial diagnostic uncertainty and the provisional diagnosis of a functional neurological disorder.

The mechanisms underlying chorea in hyperthyroidism remain poorly defined. One proposed explanation involves increased dopaminergic activity secondary to thyroid hormone excess, leading to heightened sensitivity of dopamine receptors within the basal ganglia [6,8]. This hypothesis is supported by the reversibility of symptoms following restoration of euthyroidism.

An autoimmune-mediated mechanism has also been suggested. Graves’ disease is characterized by circulating autoantibodies, and immune-mediated effects on basal ganglia function may contribute to movement disorders, even in the absence of structural abnormalities on imaging [6]. In addition, cerebral hypermetabolism associated with thyrotoxicosis may alter neuronal excitability within motor control circuits [6].

Hemichorea commonly prompts evaluation for acute cerebrovascular disease, particularly when accompanied by sensory symptoms. Due to the presentation, this patient was submitted to an extensive investigation, including magnetic resonance imaging, computed tomography angiography, electroencephalography, and cerebrospinal fluid analysis, in order to exclude structural, epileptic, infectious, and inflammatory causes. The atypical presentation and the delayed availability of thyroid function tests contributed to the initial diagnostic uncertainty and delayed diagnosis. Therefore, this case highlights the importance of including thyroid function assessment in the routine evaluation of unexplained hyperkinetic movement disorders, particularly when standard neurological investigations are non-diagnostic [2,3,6].

Management of hyperthyroidism-related chorea focuses on treatment of the underlying thyrotoxicosis. Antithyroid drugs such as methimazole inhibit thyroid hormone synthesis and represent first-line therapy [4]. Beta-adrenergic blockade may provide symptomatic relief, while corticosteroids and iodine preparations are reserved for severe cases or perioperative management [4]. In this patient, initiation of antithyroid therapy resulted in progressive normalization of thyroid function and complete resolution of choreic movements. The temporal relationship between treatment and clinical improvement supports a causal association between Graves’ disease and the movement disorder, consistent with previously reported cases [7,9,10], where a permanent remission is expected in about 50% of Graves' disease cases with treatment, compared to a spontaneous remission rate of approximately 25% [4].

## Conclusions

This case describes a patient diagnosed with Graves’ disease in whom left upper limb chorea was the initial clinical manifestation. Although hemichorea is rare in hyperthyroidism, it may be the presenting finding. Thyroid function testing should therefore be considered in patients with hyperkinetic movement disorders, particularly when neuroimaging and routine neurological investigations are unrevealing.

This case highlights an atypical presentation of Graves’ disease that contributed to a delayed diagnosis, as hyperthyroidism is not typically considered in the initial evaluation and more commonly presents with systemic symptoms. Early recognition and appropriate treatment of the underlying endocrine disorder are essential, as they may lead to rapid resolution of chorea and prevent unnecessary investigations and potentially harmful interventions. This case underlines the importance of awareness of neuroendocrine interactions and a multidisciplinary approach in patients with unexplained movement disorders.
